# Glacial lake drainage in Patagonia (13-8 kyr) and response of the adjacent Pacific Ocean

**DOI:** 10.1038/srep21064

**Published:** 2016-02-12

**Authors:** Neil F. Glasser, Krister N. Jansson, Geoffrey A. T. Duller, Joy Singarayer, Max Holloway, Stephan Harrison

**Affiliations:** 1 Department of Geography and Earth Sciences, Aberystwyth University, SY23 3DB, Wales, UK; 2 Department of Physical Geography, Stockholm University, SE-10691, Stockholm, Sweden; 3 Department of Meteorology, University of Reading, Earley Gate, PO Box 243, Reading, RG6 6BB, UK; 4 British Antarctic Survey, Madingley Road, Cambridge, UK; 5 College of Life & Environmental Science, Exeter University TR10 9EZ, Cornwall, UK

## Abstract

Large freshwater lakes formed in North America and Europe during deglaciation following the Last Glacial Maximum. Rapid drainage of these lakes into the Oceans resulted in abrupt perturbations in climate, including the Younger Dryas and 8.2 kyr cooling events. In the mid-latitudes of the Southern Hemisphere major glacial lakes also formed and drained during deglaciation but little is known about the magnitude, organization and timing of these drainage events and their effect on regional climate. We use 16 new single-grain optically stimulated luminescence (OSL) dates to define three stages of rapid glacial lake drainage in the Lago General Carrera/Lago Buenos Aires and Lago Cohrane/Pueyrredón basins of Patagonia and provide the first assessment of the effects of lake drainage on the Pacific Ocean. Lake drainage occurred between 13 and 8 kyr ago and was initially gradual eastward into the Atlantic, then subsequently reorganized westward into the Pacific as new drainage routes opened up during Patagonian Ice Sheet deglaciation. Coupled ocean-atmosphere model experiments using HadCM3 with an imposed freshwater surface “hosing” to simulate glacial lake drainage suggest that a negative salinity anomaly was advected south around Cape Horn, resulting in brief but significant impacts on coastal ocean vertical mixing and regional climate.

During the last glaciation rapid outburst floods from glacial lakes in North America into the North Atlantic affected the production of North Atlantic Deep Water (NADW) and likely caused abrupt century- to millennial-scale climate changes^1^,^2^,^3^,^4^,^5^. Although large meltwater lakes existed in the mid-latitudes of the Southern Hemisphere in Patagonia^6^,^7^,^8^, the nature of their drainage during deglaciation and the impact of this on regional climate has not been similarly investigated despite the recognition of late glacial millennial-scale climate changes in a variety of Southern Hemisphere terrestrial environments^9^. Patagonia, situated in the southern mid-latitudes near the core of the precipitation-bearing southern westerlies, is arguably one of the most climatically sensitive areas in the world and interpreting the environmental signatures recorded in marine and terrestrial records here is important to test models of environmental change and ice-sheet behaviour on regional to interhemispheric scales^10^.

The North and South Patagonian Icefields (NPI and SPI respectively; Fig. 1a) expanded and contracted in response to climatic forcing a number of times during the Quaternary^11^. At times, the two icefields coalesced to form the Patagonian Ice Sheet (PIS), which covered the Andes between latitudes 38 ^o^S and 56 ^o^S^12^,^13^. During deglaciation a series of glacial lakes formed east of the Andes in the over-deepened basins currently occupied by Lago General Carrera/Lago Buenos Aires (LGC/LBA) and Lago Cohrane/Pueyrredón (LC/LP)^6^,^7^,^8^. Previous studies^6^,^7^,^8^ have described the glacial lake sedimentology and glacial lake outline but little is known about the lake response to PIS deglaciation, especially the organization, timing and routing of drainage events. Here we present new geomorphological mapping and 16 new OSL dates that provide chronological constraints on major glacial lake nature and evolution of the region. These dates allow the first reconstruction of the glacial lake system and associated drainage routes, as well as injection sites and fluxes of meltwater pulses released to the Pacific Ocean during deglaciation of the PIS. Using numerical coupled ocean-atmosphere model simulations, our study significantly advances understanding of Southern Hemisphere cryosphere ocean/climate interactions.

The contemporary LGC/LBA lies at around 200 masl with a surface area of 1850 km^2^ and a maximum depth of ~590 m^14^. Immediately to the south, LC/LP at 155 masl, has a surface area of 325 km^2^. Both lakes are impounded at their eastern ends by large Quaternary moraines that mark the maximum extent of the PIS^15^,^16^ (Fig. 1). At its largest, Glacial Lake LGC/LBA coalesced with Glacial Lake LC/LP to form a single lake known in the literature as the “United Lake”^6^, which we refer to from now on as the Glacial Lake Patagonian Ice Sheet. As the PIS receded and split into the NPI and SPI this lake drained finally westward through the Andes into the Pacific Ocean via the Rio Baker into the Golfo Peñas (47^o^15′25″S, 74^o^50′25″W; Fig. 1).

Our new mapping of the glacial geomorphology and elevation distribution of perched deltas, beaches and shorelines indicates that Glacial Lakes LGC/LBA and LC/LP were characterised by five main Glacial Lake Stages (named here Stages A to E on Fig. 1b with sample sites and geomorphological context shown in Fig. 1c and lake evolution stages indicated on Fig. 2). Perched deltas, beaches and shorelines formed by the glacial lakes show clear peaks in elevation bands that allow us to identify former lake levels (Fig. 1b). In optimal settings we would expect shoreline features to occupy narrow elevation bands but the situation at deglaciation, with non-uniform regional isostasy and the successive drainage of the glacial lakes eastward due to moraine failure, created both sloping shoreline features and relatively steep delta surfaces^6^. We regard the broad distribution of shoreline features within individual peaks to be an effect of these processes.

Glacial lakes pre-dating the formation of Glacial Lakes LGC/LBA and LC/LP formed in the Chacabuco River valley (Early Glacial Lake Stage A). Delta features as high as 700 and 550 m a.s.l. respectively indicate the existence of these lakes. Glacial Lake Chacabuco existed as an isolated lake at around 620 m a.s.l. with a col at the headwaters of Rio Ghio (C-1) before it drained southward towards Puesto Tejuela into Glacial Lake LC/LP (Ds-1), which successively drained eastward into the head waters of Rio Blanco (Ds-2) through moraine failure^17^. Glacial Lake LGC/LBA also drained eastward (Ds-3) due to moraine failure. During ice-marginal recession Glacial Lake LC/LP drained into the Glacial Lake LGC/LBA basin to form the Glacial Lake Patagonian Ice Sheet (Glacial Lake Stage B). Lake elevation was still controlled by the integrity of the eastern moraines. No OSL dates for these lake stages were obtained but moraines dated by TCN to 13.2 and13.8 kyr^12^,^28^ near the NPI provide a constraint on the age of this lake.

Glacial Lake Stage B marks the growth of the Glacial Lake Patagonian Ice Sheet at 480 m a.s.l. (Fig. 1c). Glacial Lake LC/LP drained during ice-marginal retreat through canyons towards the Puerto Guada east of Lago Bertrand (Ds-4). Ice-marginal positions, dated by TCN between 11.1 and 11.7 kyr^12^, indicate the age of this glacial lake. The cluster of OSL dates (Fig. 1c) also fits well with the suggested lake outline at this time. The “United Lake” successively lowered from 480 to 380 m a.s.l. by moraine degradation east of the town of Perito Moreno (C-2). Drainage was eastward into the Rio Deseado catchment Ds-5). The lake seems to have been stabilized by its adjustment to a col at 380 m a.s.l.

During Glacial Lake Stage C the PIS had receded sufficiently to allow the first westward drainage event. As the ice margin receded, a pathway through the canyon at Lago Tranquilo was exposed (Ds-6). This col at 350 m a.s.l. dictated the outline of the lake during Glacial Lake Stage C.

During Glacial Lake Stage D the lake level dropped from 350 to 315 m a.s.l. (Ds-7) as the PIS receded further and drainage took place at a lower elevation through the col at 315 m a.s.l. at the head waters of Rio Bravo north of Villa O’Higgins (C-4). The existence of fragments of shoreline features at 330 m a.s.l. indicate the possible existence of an intermediate glacial lake stage. This could mark progressive erosion of the col or fluctuations in the position of the ice margin of the PIS. During Glacial Lake Stage E, after the adjustment to the 260 m a.s.l. level (Ds-8) set by the col, drainage of water was southward into Rio Pascua and from there into Lago O’Higgins (C-5). The final drainage of Glacial Lake NPI occurred westward into the Golfo Peñas (Ds-9) at the final separation of the PIS into the NPI and SPI.

Glacial Lake Stage A (620 to 480 m a.s.l.) is dated by ice-marginal positions from Terrestrial Cosmogenic Nuclide (TCN) dates at Lago Tranquilo, Lago Negro and at the Leones and Chacabuco valley mouths^18^,^19^, indicating glacier recession between 13.8 kyr and 11.5 kyr. Before the transition into Glacial Lake Stage B, marked by the formation of Glacial Lake NPI at 380 masl, shoreline features formed above 380 masl. These are constrained by OSL samples LTH01 (11.8 ± 1.2 kyr), collected at 452 masl from a section in palaeo-beach sands and gravels at Los Tres Hermanos and MCS02 (12.0 ± 1.3 kyr), collected at 444 masl from aeolian sand capping a c 5 m-wide paleo-beach at Las Mercedes. The age of the 370-440 masl surface is ~11 kyr based on the weighted mean of three samples; BC02 (12.9 ± 2.1 kyr), collected at 396 masl from a section in the shoreface platform of a delta fragment in the col at Bertrand; PII02 (11.1 ± 4.2 kyr), collected at 396 masl from a section in delta front foresets near Puerto Ibáñez and RII01 (10.3 ± 0.9 kyr), collected from the upper surface of a flat-topped feature at Río Ibáñez. Full details are presented in the Supplementary Information.

No ages were obtained for Glacial Lake Stage B. One reason for this could be that this stage only existed for a short period before the drainage route over the Lago Tranquilo col opened at 350 masl. TCN dates^18^,^19^ indicate an advanced Glacier Exploradores at ~10 kyr supporting the existence of a damming ice margin west of Lago Tranquilo at the time of Glacial Lake Stage C. Several OSL dates support the existence of glacial lakes at levels below 350 masl. An age of 8.0 ± 0.5 kyr is given from aeolian sand capping a large flat-topped delta at Puente Santa Marta at 333 masl (Sample PSM01) and confirms that the glacial lake had drained by this time and subaerial aeolian deposition was taking place. Sample MCS01 (8.5 ± 0.9 kyr), collected at 330 masl from a section in palaeo-beach sands and gravels at Las Mercedes, provides a similar age. We suggest that these youngest dates mark the onset of routing events SE of the NPI and a drop in lake level from 350 to 260 masl (Fig. 2).

Our new OSL dates for the timing of lake drainage have important implications for understanding Patagonian glacial chronologies. Outlet glaciers from the east of the NPI were still expanded at ~11 kyr, before receding rapidly after ~10 kyr, possibly triggered by enhanced lake-calving. This picture of an expanded NPI around ~11 kyr is supported by TCN dating of boulders on moraines at the mouths of the Nef, Leones and Colonia Valleys, east of the NPI, which indicate that outlet glaciers advanced, or at least stabilised, to form large moraines at 11.0 ± 0.5/11.2 ± 0.6, 11.5 ± 0.6, 11.7 ± 0.6 and 12.8 ± 0.7 kyr^19^. Final westward drainage of the Glacial Lake NPI around 8.5 kyr marks the separation of the PIS into the NPI and SPI. Our OSL date for this event is much later than the 12.8 kyr previously suggested for this separation^6^.

At its maximum extent, we calculate that the lake had a surface area of ~7400 km^2^ and an estimated volume of ~1500 km^3^. The volume of water released during these drainage events was between 160 km^3^ and 1150 km^3^. Palaeo-lake levels were controlled by the elevation of potential overspill routes through topographic low points and cols, whose availability was in turn determined in the west by the position of the ice margin, and in the east by moraine dams (Figs 1 and 2).

We used an AOGCM (HadCM3) to model the impact of glacial lake drainage on the regional climate and oceanic processes. The influx of freshwater from westward drainage occurred at a location where the prevailing coastal currents turn southwards towards the Antarctic Circumpolar Current (ACC), rather than following the Peru coastal current northward and the model response is highly sensitive to the exact location of the meltwater input (see Supplementary Information). In our simulation the mixed layer depth generally decreases following the advection of freshwater around Cape Horn (Fig. 3 upper panel), as a result of increased vertical stability due to lower density surface waters. The reduction of the mixed layer depth is greatest in austral winter, and the impact on climate is also largest at this time of year. There is an increase in eastward surface current velocity around the coast, possibly enabled by reduced mixing. Cooler surface temperatures occur directly over the location of the hosing influx and around Cape Horn (Fig. 3 lower panel). The temperature and salinity anomalies are advected into the Malvinas current and impact the location where the cold northward Malvinas current meets the southward flowing warm Brazil current. This results in a year-round dipole temperature anomaly at the Brazil-Malvinas confluence. The lower surface temperatures experienced in austral winter result in lower evaporation rates and reduction in winter precipitation of up to 10% over the region of freshwater influx and the southern tip of South America. The duration of the impact of the influx of freshwater is short-lived. Unlike the potentially catastrophic consequences of freshwater input into the North Atlantic, the amount of freshwater is relatively small; there is no overturning in this region of the ocean, and the salinity anomalies are quickly entrained into the ACC. Following a freshwater influx of one year, the ocean surface salinities returned to normal in the subsequent five years (Fig. S5).

Our new data, including chronologically well-constrained meltwater events, have the potential to increase our understanding of the role of glacial lakes in the climatic system^20^,^21^,^22^. This is supported by recent research on eastern South Pacific proxy data which stresses the important relationship between the thermohaline circulation and meltwater pulses released during the deglaciation of the Patagonian Icefields^23^,^24^. The melting of the Patagonian Icefields acted as a trigger for the collapse of buoyancy forcing and ventilation and subsequent deglacial readjustment of the ventilation process in the eastern South Pacific. These previous conclusions were, however, based on the assumption of an average deglacial meltwater flux from the icefields and we have shown that this assumption is open to question. If the meltwater signal from the Patagonian Icefields described here can be quantified and isolated in proxy records, then the driving mechanisms for millennial-scale climate variations in this region of the SH will come more clearly into focus^25^,^26^,^27^.

## Methods

Geomorphological features associated with the former glacial lakes including perched deltas, palaeo-shorelines, palaeo-beaches and lake overspill/drainage cols were mapped from remotely sensed images and in the field^12^,^28^. Remotely sensed images include high resolution imagery provided by Google Earth, Landsat 7 ETM + scenes (30 metre spatial resolution) and ASTER (15 metre spatial resolution). Satellite images were overlain on a Digital Elevation Model (DEM) based on 90 m cell size Shuttle Radar Topography Mission (SRTM) data to identify cols and other lake drainage overspill routes. All mapped features were stored in ArcMap GIS software, which was also used to calculate lake areas and volumes. Elevation data for shoreline features were extracted after a 30 meter buffering procedure of individual features to create histogram plots of the elevation distribution. In the field, lake elevations were surveyed using a decimetre-precise Trimble Net RS dGPS. All luminescence measurements were performed in the Aberystwyth Luminescence Research Laboratory using previously described procedures^29^. The single aliquot regenerative dose (SAR) protocol was used for equivalent dose (D_e_) measurement. Potential effects on regional ocean circulation were investigated using the UK Hadley Centre global climate model, HadCM3. HadCM3 consists of coupled atmospheric model, ocean model and sea ice model components^30^,^31^. The resolution of the atmospheric model is 2.5° in latitude by 3.75° in longitude by 19 unequally spaced vertical levels. Further information on all methods is provided in the Supplementary Information.

## Additional Information

**How to cite this article**: Glasser, N. F. *et al*. Glacial lake drainage in Patagonia (13-8 kyr) and response of the adjacent Pacific Ocean. *Sci. Rep*. **6**, 21064; doi: 10.1038/srep21064 (2016).

## Supplementary Material

Supplementary Information

## Figures and Tables

**Figure 1:**
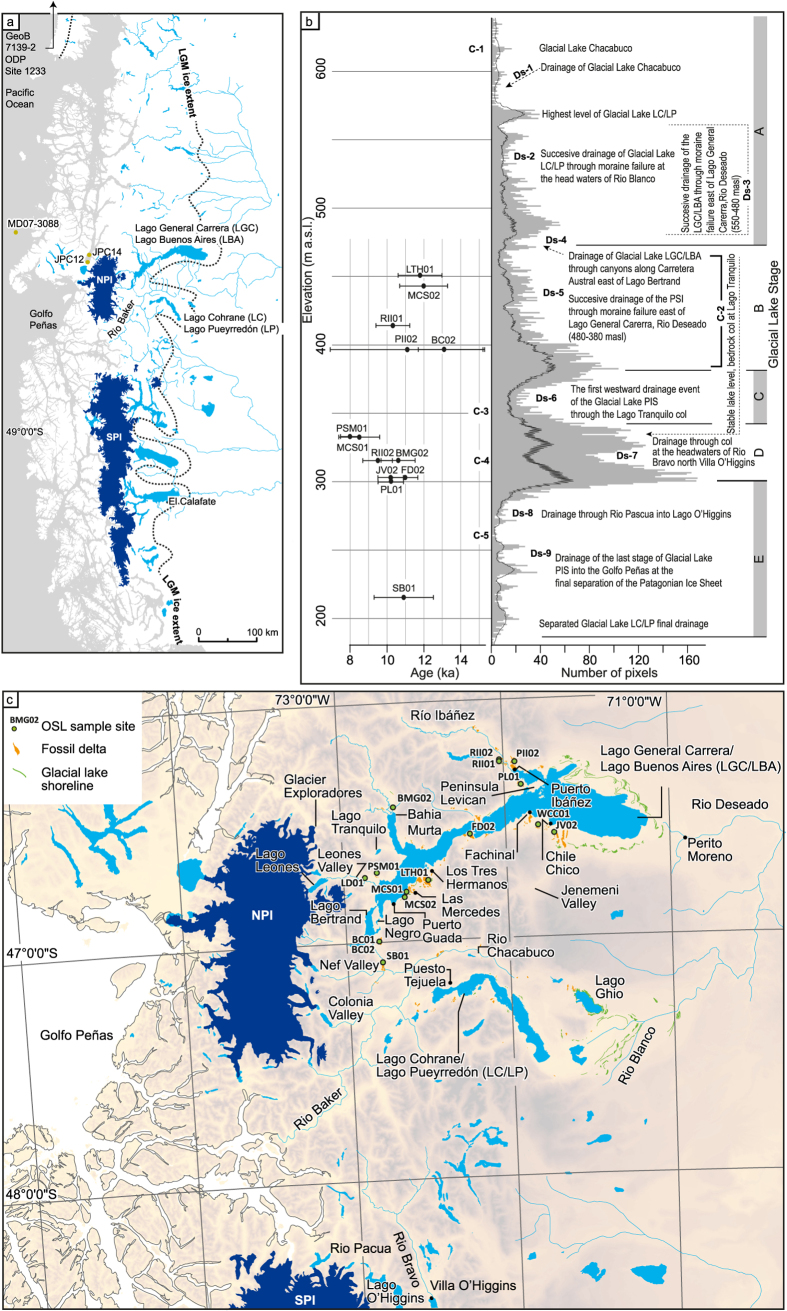
(**a**) Southern South America with the location of the Lago General Carrera/Lago Buenos Aires and Lago Cohrane/Pueyrredón basins. The Last Glacial Maximum (LGM) extent of the former Patagonian Ice Sheet and the contemporary North and South Patagonian Icefields (NPI and SPI) are indicated. Details of offshore core locations are discussed in the Supplementary Information. (**b**) Elevation distribution of glacial lake shoreline features (in grey) generated by pixel-counting in a Digital Elevation Model. To generate these data we mapped all glacial lake shoreline features and counted the number of pixels occupied by the features. The higher the pixel count, the more widespread the feature. Solid black line shows the running mean. Glacial Lake Stages are indicated by letters A to E. Analysed OSL dates with error bars are presented on the left hand side of the panel at their correct elevation. Label C-1 to C-5 show the position of bedrock cols and Labels Ds-1 to Ds-9 indicate lake drainage sites. (**c**) Geomorphological evidence for the extent of the former glacial lakes, place names mentioned in the text and the locations of OSL samples dated in this study. The geomorphological evidence is a combination of previous mapping of glacial landforms^28^ and new mapping of features related to the former glacial lakes. All maps were created in ArcGIS (ArcMap 10.2.2; http://www.esri.com/software/arcgis).

**Figure 2:**
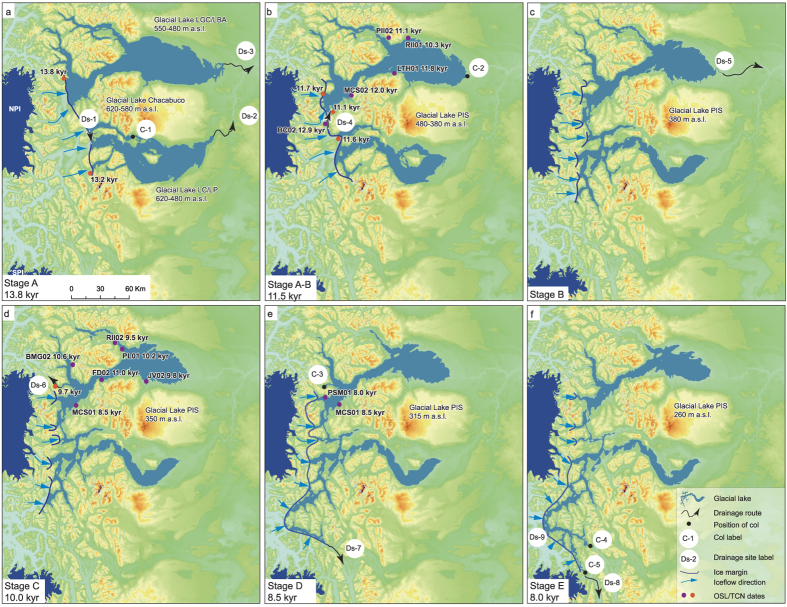
Former glacial lake outlines draped on SRTM shaded relief indicating the patterns of drainage during Glacial Lake Stages A to E. The contemporary North Patagonian Icefield (NPI) and South Patagonian Icefield (SPI) are indicated. New OSL dates obtained in this study and relevant TCN dates^19^ are marked. Important topographic cols C-1 to C-5 and major lake drainage sites Ds-1 to Ds-9 are indicated. The former ice margin of the expanded Patagonian Ice Sheet that dammed the lakes is also indicated. Panels (**a,c)** indicate different time periods in lake initiation and coalescence with an ice dam impeding drainage at the western end of the lakes and lake elevation determined by the moraine dams at the eastern ends of the lakes. Panel (**d**) indicates ice recession as a drainage route opens up at the western end of the lake through the col at Lago Tranquilo and the lake drained rapidly westward. Panel (**e**) shows the situation as further ice recession opened up lower elevation drainage routes to the SE of the NPI and the lake drained southeastward at the head waters of Rio Bravo north of Villa O’Higgins. Panel (**f)** shows lake drainage during the final stages of deglaciation into Rio Pascua and Lago O’Higgins. After this stage the final drainage of the glacial lake occurred at the final separation of the Patagonian Ice Sheet. See text for full explanation. All maps were created in ArcGIS (ArcMap 10.2.2; http://www.esri.com/software/arcgis).

**Figure 3:**
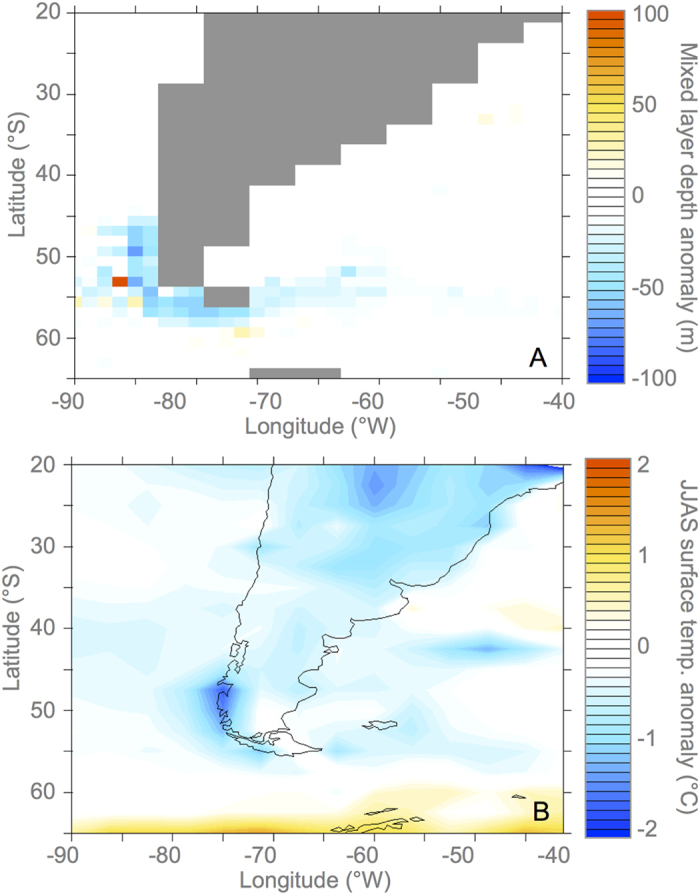
Results of climate modelling experiments showing ocean response to addition of freshwater at the glacial lake drainage site. The upper panel shows the annual mixed layer depth anomaly, averaged over five years from the simulations YEAR5S-CNTL. The lower panel shows the austral winter (JJAS) surface temperature anomaly from YEAR5S-CNTL. Grey box indicates location of Brazil-Malvinas Confluence. Maps were created using Ferret; a product of NOAA’s Pacific Marine Environmental Laboratory (available at http://ferret.pmel.noaa.gov/Ferret/).
