# Supplementary material for: Glacial lake drainage in Patagonia (13-8 kyr) and response of the adjacent Pacific Ocean
Source: Sci Rep. 2016 Feb 12;6:21064. doi: 10.1038/srep21064 (PMC4751529; doi:10.1038/srep21064)
Supplement: Supplementary Information [file srep21064-s1.pdf]

Glacial lake drainage in Patagonia (13-8 kyr) and response of the adjacent Pacific Ocean

Neil F. Glasser, Krister N. Jansson, Geoffrey A.T. Duller, Joy Singarayer, Max Holloway and Stephan Harrison

SUPPLEMENTARY INFORMATION

Further background information and regional setting

Fragments of paleo-shorelines and paleo-beaches occur at many elevations above the contemporary lakes in the Lago General Carrera/Lago Buenos Aires (LGC/LBA) and Lago Cohrane/Pueyrredón (LC/LP) basins (Figures S1 and S2). Large sediment accumulations, interpreted by Bell (2008; 2009) as lacustrine braid deltas, occupy the mouths of almost every tributary valley around LGC/LBA and define these former ice-dammed lake levels (Figure S3). The rapid abandonment of these deltas indicates that lake drainage was abrupt (Bell, 2008). According to Turner et al. (2005) the former LGC/LBA lake levels are at 305 to 355 m and 390–484 m, with a higher level at 489–512 m in the southern basin and valleys of the LC/LP catchment. Turner et al. (2005) also recognised a higher elevation shoreline at 633 to 654 m, which is restricted to local valleys and slopes surrounding the catchment of LC/LP. In the main paper, we provide the altitudes of the former lakes as a range because there is East/West regional tilt in shoreline and delta elevation caused by past and ongoing differential post-glacial isostatic recovery (Ivins and James, 1999; 2004; Chen et al. 2007). Elevations on former lake levels rise towards the former ice centre because post-glacial isostatic uplift has been greatest here.

In the main paper we define former moraine-dammed and ice-dammed lakes by perched deltas, palaeo-beaches and palaeo-shorelines at three major levels: 300-320 masl, 370-440 masl and 460-520 masl (Figure S3). The highest level lake (460-520 masl) was relatively stable and drained slowly through moraines to the east between 11 and 12 kyr, but the 300 masl and the 370-440 masl lakes drained rapidly westward though the Andes at 11 and 10 kyr. Lake drainage was controlled by west-flowing drainage routes through cols at Lago Tranquilo (not recognised in earlier studies) and the mouth of the Nef Valley in the Rio Baker that opened up as the Patagonian Ice Sheet receded and decoupled to form the NPI and SPI.

Further information on methods

OSL dating (Tables S1 & S2)

Sediment samples for OSL dating were obtained by inserting an opaque plastic tube into cleaned sections, avoiding obvious weathering horizons and other pedogenic features. Sample tubes were removed from the sections, the ends packed with plastic bags to ensure that sample mixing did not occur during transport, wrapped in opaque plastic bags and sealed with tape to protect from light. All luminescence measurements were performed in the Aberystwyth Luminescence Research Laboratory using procedures based on those described in Duller (2006). For OSL measurements sand-sized quartz grains approximately 200 µm in diameter were separated. Carbonates were removed using HCl, organic material using H_2O_2, and

heavy minerals and feldspars were removed on the basis of density using solutions of sodium polytungstate at 2.62 and 2.70 $g.cm^{-3}$. Further purification was achieved by immersing the grains in 48% hydrofluoric acid for 40 minutes, etching the outer 10µm from the surface of the quartz grains, and removing any remaining feldspars. All luminescence measurements were made on single grains in order to maximize the opportunity of identifying the grains within a sample that had had their OSL signal reset at the time of deposition (Duller 2008). A Risø TL/OSL-DA-15 equipped with a focused laser system for single grain OSL measurements (Bøtter-Jensen et al., 2003) was used for all samples. The environmental dose rate to samples was calculated using a combination of thick source alpha counting and beta counting. The contribution from cosmic rays was evaluated using the equations provided by Prescott and Hutton (1994).

We collected samples from a range of landforms around the former lake margin (Fig 1C of the main paper). Seven samples for luminescence dating were obtained from the 300-320 masl lake level, five samples from the 370-440 masl lake level, two samples from the 460-520 masl lake level and one sample from a modern source bordering dune adjacent to a modern delta.

The single aliquot regenerative dose (SAR) protocol was used for equivalent dose (D_e) measurement. Samples were given a preheat of 220°C for 10 seconds after each regeneration dose and a cut heat of 160°C after each test dose (typically ~15 Gy). Acceptance criteria included a recycling ratio within 10% of unity, OSL signal at least 3 times greater than the uncertainty in the background, an uncertainty of less than 20% on measurement of the test dose, and an OSL-IR depletion ratio within 10% of unity (Duller 2003).

Dose recovery experiments were undertaken on three samples to assess the ability of the SAR protocol to accurately measure a known laboratory radiation dose. Grains of quartz were bleached at room temperature for 100 seconds using blue LEDs (470 nm, $35mW.cm^{-2}$), left for 10ks to allow charge in the 110°C TL peak to escape and then the bleaching procedure repeated a second time. A dose similar to the expected D_e was then given to the grains. All three samples were able to recover the given dose within the 10% limits conventionally adopted for such a test, confirming that the SAR protocol can measure a known radiation dose (Tables S1). The overdispersion (σ_d) of the data sets varies from 14% to 26% respectively. This is the scatter in the data that cannot be accounted for by measurement uncertainty and counting statistics. The cause of this scatter is unclear. In the analysis above, a value of 20% has been used for the maximum uncertainty allowed in the determination of the test dose. This is double the value that is normally used and was designed to allow incorporation of data from a larger number of grains in order to help constrain the age models. To test whether this relaxed criteria was inadvertently leading to high overdispersion the dose recovery data were re-analysed using a test dose threshold of 10%. For all samples there was no detectable change in either the value of overdispersion, or the ability to recover the known dose. However in all cases the number of grains accepted fell by 70%. The cause of the high overdispersion in D_e values for quartz from this region is not known.

The majority of sediments analysed in this study are from deltas associated with the former lake, and the luminescence signal in the grains is likely to have been incompletely bleached at deposition. Statistical analysis of the dose distributions to isolate the population of grains which was bleached at deposition followed the method described by Duller (2006). This approach used the finite mixture model (FMM) and followed the work of Rodnight et al. (2006). The validity of this approach was demonstrated by Duller (2006) by comparing the OSL ages for a number of glacio-fluvial sediments with independent age control. A small number of samples in this study were collected from beach or from aeolian facies and for these samples the central age model (CAM) was used. Figure S4 shows the D_e distributions measured for the samples. A summary of the luminescence data for all the OSL samples is given in Table S2, including the dosimetry data, the number of quartz grains analysed, the D_e used for age calculation, and the calculated ages.

Climate modelling experiments

Potential effects on regional ocean circulation were investigated using the UK Hadley Centre global climate model, HadCM3. HadCM3 consists of coupled atmospheric model, ocean model and sea ice model components, (Pope et al., 2000; Gordon et al., 2000). The resolution of the atmospheric model is 2.5° in latitude by 3.75° in longitude by 19 unequally spaced vertical levels. The spatial resolution over the ocean in HadCM3 is 1.25° by 1.25° by 20 unequally spaced layers in the ocean extending to a depth of 5200 m. The model contains a range of parameterizations, including a detailed radiation scheme that can represent the effects of minor trace gases (Edwards and Slingo, 1996). The ocean model uses the Gent–McWilliams mixing scheme (Gent and McWilliams, 1990). The sea ice model is a simple thermodynamic scheme and contains parameterizations of ice drift based on surface ocean currents (Cattle and Crossley, 1995). The model version used here incorporates the MOSES1 land surface scheme (Cox et al., 1999) and fixed vegetation. HadCM3 was forced with prescribed changes in orbit (altering the seasonal and latitudinal distribution of solar insolation), greenhouse gases, sea level, and ice-sheet evolution appropriate to 10 kyr BP. For further details on these boundary conditions see Singarayer and Valdes (2010). We performed three simulations: (1) a control simulation (CNTL) had 10 kyr BP boundary conditions and no freshwater hosing, (2) 500,000 km^3 freshwater added to the west coast of Patagonia (Fig S5) over a 1-year "hosing" simulation (YEAR1S), (3) the same hosing rate as (b) but over a 5-year period (YEAR5S); this was to enable further statistical evaluation of the climatic impact.

Further details of the sedimentology, geomorphology and OSL sample context

Perched deltas here are typically flat-topped, and a well-developed shoreline indicates that the lake level was stable for a considerable length of time at this elevation before falling abruptly (Fig. S1). Numerous, closely spaced shorelines and associated sloping deltas indicate that this lake level lowered more or less continually throughout its existence and was not stable at this level for any length of time. In all cases, sediments are typically coarse gravels and sands commonly containing 1-2 m thick medium to fine sand lenses (Fig. S2). Rounded and well-rounded clasts dominate the sediments. Sections are typically capped by massive fine sand, interpreted as a wind-blown deposit.

A summary of the glacial lake stages is presented in Fig. 2 of the main paper and Table S3.

Glacial stage A-B (Fig. 2 of main paper)
This glacial lake stage is constrained by sample LTH01 (11.8 ± 1.2 ka), collected at 452 masl from a section in palaeo-beach sands and gravels at Los Tres Hermanos. Sample MCS02 was collected at 444 masl from aeolian sand capping a c 5m-wide paleo-beach at Las Mercedes. It would be expected that this sample would be well bleached. The central age model (CAM) for the sample gives an age of 6.5 ± 0.7 ka, and indicates that the lake had drained by this time and subaerial aeolian deposition was taking place. However, the distribution of D_e values for this sample is broader than would be expected for an aeolian sediment. No exposure was present in this palaeo-beach and the sample was collected from a pit dug to a depth of 0.5m. Because the sampling depth is very shallow, it is possible that the broad distribution of D_e values results from the introduction of younger grains by bioturbation. A distinct population of older grains can be observed in the sample and these give an age of 12.0 ± 1.3 ka, which is far closer to the age obtained from LTH01. This glacial lake stage is also constrained by BC02 (12.9 ± 2.1 kyr), collected at 396 masl from a section in the shoreface platform of a delta fragment in the col at Bertrand; RII01 (10.3 ± 0.9 kyr), collected from a shallow pit excavated on the upper surface of a flat-topped feature at Río Ibáñez; and PII02 (11.1 ± 4.2 kyr), collected at 396 masl from a section in the delta front foresets in one of a staircase of steeply dipping deltas near Puerto Ibáñez. Sample WCC01 (16.8 ± 1.5 kyr) was collected at 403 masl from a section in the shoreface platform of a delta fragment west of Chile Chico but is poorly bleached so is not included in this calculation of the mean age. A further sample (BC01) was collected at 396 masl from a section in the shoreface platform of a delta fragment in the col at Bertrand but yielded insufficient data to calculate a reliable age.

Glacial lake stage C (Fig. 2 of main paper)

The elevation of lake levels above the Lago Tranquilo col at 350 masl was controlled by the moraines at the eastern end of LGC/LBA and LC/LP, and the level of this lake fell slowly as the lake outlet channel progressively incised to form an outflow through the moraines. The lake initially drained to a level controlled by a rock-cut channel cut into a col at 350 masl at Lago Tranquilo in the Rio Bayo Valley and not via the Rio Baker as previously suggested (Turner et al., 2005). This lake level was stable because the col at Lago Tranquilo remained ice-free during deglaciation.

The final drainage route and the lowering of the lake level below 260 masl occurred through the Rio Baker valley. A younger age from aeolian sand capping a large flat-topped delta at Puente Santa Marta at 333 masl (Sample PSM01; 8.0 ± 0.5 kyr) indicates that the lake had drained by this time and subaerial aeolian deposition was taking place.

Glacial lake stage D and E (Fig 2 of main paper)
As the ice receded into the Nef valley, a third drainage route for the 330-260 masl paleo-lake developed southeastward through the Rio Bravo and Pascua systems. Dates that constraine this glacial lake stage are: Samples PL01 (10.2 ± 0.7 kyr) and

RII02 (9.5 ± 0.8 kyr) collected at 301 masl and 315 masl from sections in the shoreface platform of large flat-topped deltas on Peninsula Levican and the Rio Ibanez respectively. Sample MCS01 (8.5 ± 0.9 kyr), collected at 330 masl from a section in palaeo-beach sands and gravels at Las Mercedes, provides a similar, but slightly younger, age. Sample FD02 (11.0 ± 0.7 kyr) was collected at 303 masl from a section in the shoreface platform of the delta at Fachinal. This sample is important because here there is independent age control from cosmogenic isotope dates of boulders on moraines built on top of the delta (Douglass et al., 2005). The cosmogenic isotope dates indicate that local mountain glaciers advanced onto the delta surface to build moraines at ca. 8.5 and 6.2 kyr so the delta itself must be older than these ages, which is indeed the case. Sample BMG02 (10.6 ± 0.9 kyr) was collected at 315 masl from a section in the delta front foresets in a large flat-topped delta at Bahia Murta. Sample JV02 (9.8 ± 0.7 kyr) was collected at 303 masl from a section in the shoreface platform of a delta fragment in the Jenemeni Valley.

Supplementary Figures

Fig S1. Photographs of key landforms relating to the ice-dammed lakes. A. Multiple lake delta surfaces at Puerto Ibáñez. B. Valley-mouth deltas at Rio Claro. C. Clearly defined depositional shorelines (arrowed) at un-named site near Lago Negro. D. Lake shorelines forming erosional notches (arrowed) in bedrock above Lago Negro.

Fig S2. Photographs of key sediments relating to the ice-dammed lakes. A. Section in ~100m high steep delta front at Bahia Murta. Figure (circled) standing on talus slope for scale. The upper ~15m is in situ but lower slope is talus. B. Rounded cobble gravel interpreted as a beach facies in un-named roadside cutting neat Las Mercedes. Trowel (circled) for scale. C. parallel-laminated coarse sands and gravel in delta near Puerto Bertrand. Trowel (circled) for scale. D. Erosional unconformity in delta sands and gravels in un-named quarry near the mouth of the Jenemeni Valley. Figure (circled) for scale. E. Example of detailed sample context for OSL dating, with sample collection in a sand lense.

Fig S3. Cross-section of a typical delta fan showing the subaerial delta top and the subaqueous delta front (divided into a shoreface platform and delta front slope). Circles 1 to 3 indicate different OSL sampling contexts discussed in the text of the main paper. Diagram modified from Figure 10 in Bell (2009).

Fig S4. Radial plots of equivalent dose data for Optically Stimulated Luminescence samples collected from former lake levels around the shores of Lago General Carrera/Lago Buenos Aires and Lago Cohrane/Pueyrredón.

Fig. S5. Further climate modelling experiment results. The upper panel shows the location of hosing from glacial lake drainage in black; the lower panel shows a time series of surface ocean salinity averaged over 290° to 300°W and 60° to 45°S once the YEAR1S hosing was stopped, indicating the time taken to return to normal conditions.

Supplementary Tables

Table S1. Dose recovery data using the OSL signal from single grains of quartz.

Table S2. Analytical data associated with OSL dating from samples of paleo-beaches and deltas above Lago General Carrera/Lago Buenos Aires.

Table S3. Summary of the evidence for former lake levels of Lago General Carrera/Lago Buenos Aires. Former lake levels are based on dGPS elevations collected in the field for geomorphological features including perched deltas, paleo shorelines, terraces, beaches and drainage cols. Lake area, lake volume and drainage volume were calculated by overlaying former lake elevations onto SRTM data in Arc-Map GIS. Stages A to E correspond to stages A to E in Figure 2 of the main paper.

Supplementary References

Bell, C.M. 2008. Punctuated drainage of an ice-dammed Quaternary lake in Southern South America. *Geografiska Annaler* 90A, 1-17.

Bell, C.M. 2009. Quaternary lacustrine braid deltas on Lake General Carrera in southern Chile. *Andean Geology* 36(1), 51-65.

Bøtter-Jensen, L., Andersen, C.E., Duller, G.A.T. and Murray, A.S. (2003). Developments in radiation, stimulation and observation facilities in luminescence measurements. *Radiation Measurements* **37**(4-5): 535-541.

Cattle, H., Crossley, J., 1995. Modelling Arctic climate-change. *Philos. Trans. Royal Soc*. A 352, 201–213.

Chen, J. L., Wilson, C. R., Tapley, B. D., Blankenship, D. D. and Ivins, E. R. 2007. Patagonia Icefield melting observed by Gravity Recovery and Climate Experiment (GRACE). *Geophysical Research Letters* 34, L22501, doi:10.1029/2007GL031871.

Cox, P.M., Betts, R.A., Bunton, C.B., Essery, R.L.H., Rowntree, P.R., Smith, J., 1999. The impact of new land surface physics on the GCM simulation of climate and climate sensitivity. *Clim. Dyn* 15, 183–203.

Douglass, D.C., Singer, B.S., Kaplan, M.R., Ackert, R.P., Mickleson, D.M., and Caffee, M.W., 2005. Evidence of early Holocene glacial advances in southern South America from cosmogenic surface-exposure dating. *Geology* 33, 237-240.

Duller, G.A.T., 2003. Distinguishing quartz and feldspar in single grain luminescence measurements. *Radiation Measurements* 37, 161-165.

Duller, G.A.T. 2006. Single grain optical dating of glacigenic sediments. *Quaternary Geochronology* **1**: 296-304.

Duller, G.A.T. 2008. Single grain optical dating of Quaternary sediments: why aliquot size matters in luminescence dating. *Boreas* **37**: 589-612.

Edwards, J.M., Slingo, A., 1996. Studies with a flexible new radiation code.1. Choosing a configuration for a large-scale model. Q.*J. Royal Met. Soc* 122, 689–719.

Gent, P.R., McWilliams, J.C., 1990. Isopycnal mixing in ocean circulation models. *J. Phys. Oceanogr* 20, 150–155.

Gordon, C., Cooper, C., Senior, C.A., Banks, H., Gregory, J.M., Johns, T.C., Mitchell, J.F.B., Wood, R.A., 2000. The simulation of SST, sea ice extents and ocean heat transports in a version of the Hadley Centre coupled model without flux adjustments. *Clim. Dyn.* 16, 147–168.

Ivins, E. and T. James, 1999. Simple models for late Holocene and present-day Patagonian glacier fluctuations and predictions of a geodetically detectable isostatic response. *Geophys. J. Int., 138*: 601-624.

Ivins, E. R., and T. S. James 2004. Bedrock response to Llanquihue Holocene and present-day glaciation in southernmost South America. *Geophys. Res. Lett*., 31, L24613, doi:10.1029/2004GL021500.

Pope, V.D., Gallani, M.L., Rowntree, P.R., Stratton, R.A., 2000. The impact of new physical parameterisations in the Hadley Centre climate model: HadAM3. *Clim. Dyn.* 6, 123–146.

Prescott, J.R. and Hutton, J.T. 1994. Cosmic ray contributions to dose rates for luminescence and ESR dating: large depths and long-term time variations. *Radiation Measurements* **23**: 497-500.

Rodnight, H., Duller, G.A.T., Wintle, A.G., Tooth, S., 2006. Assessing the reproducibility and accuracy of optical dating of fluvial deposits *Quaternary Geochronology* 1, 109-120.

Turner, K.J., Fogwill, C.J., McCulloch, R.D., and Sugden, D.E., 2005, Deglaciation of the eastern flank of the North Patagonian Icefield and associated continental-scale lake diversions: *Geogr. Ann*., 87A, p.363-374.

Singarayer, J.S., and Valdes, P.J., 2010. High-latitude climate sensitivity to ice-sheet forcing over the last 120 kyr. *Quat. Sci. Rev*., 29, 43-55.

Figure S1

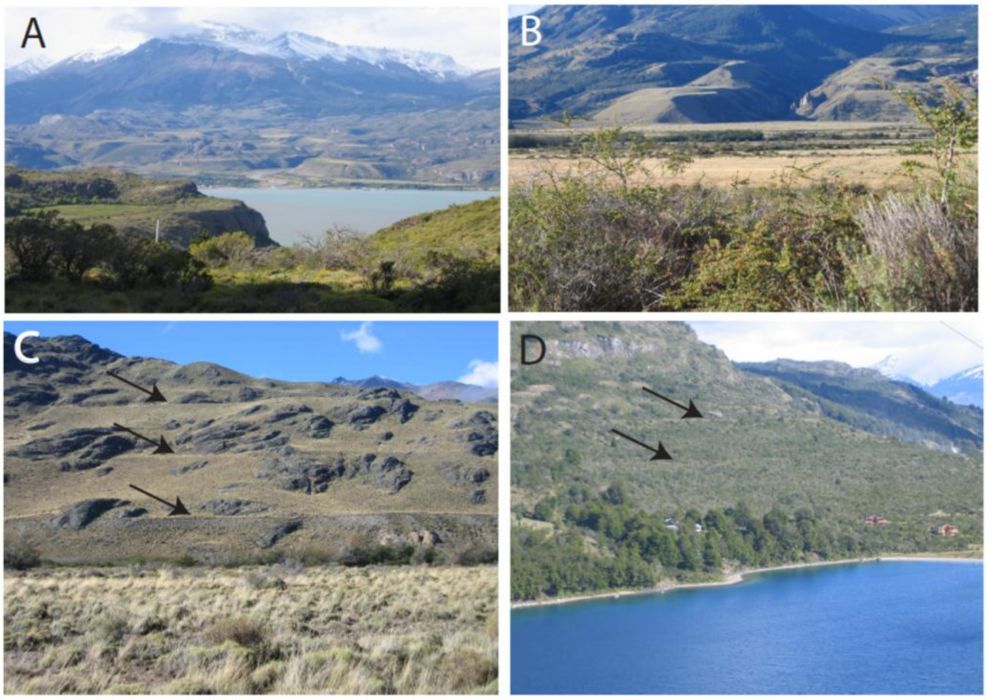

Figure S2

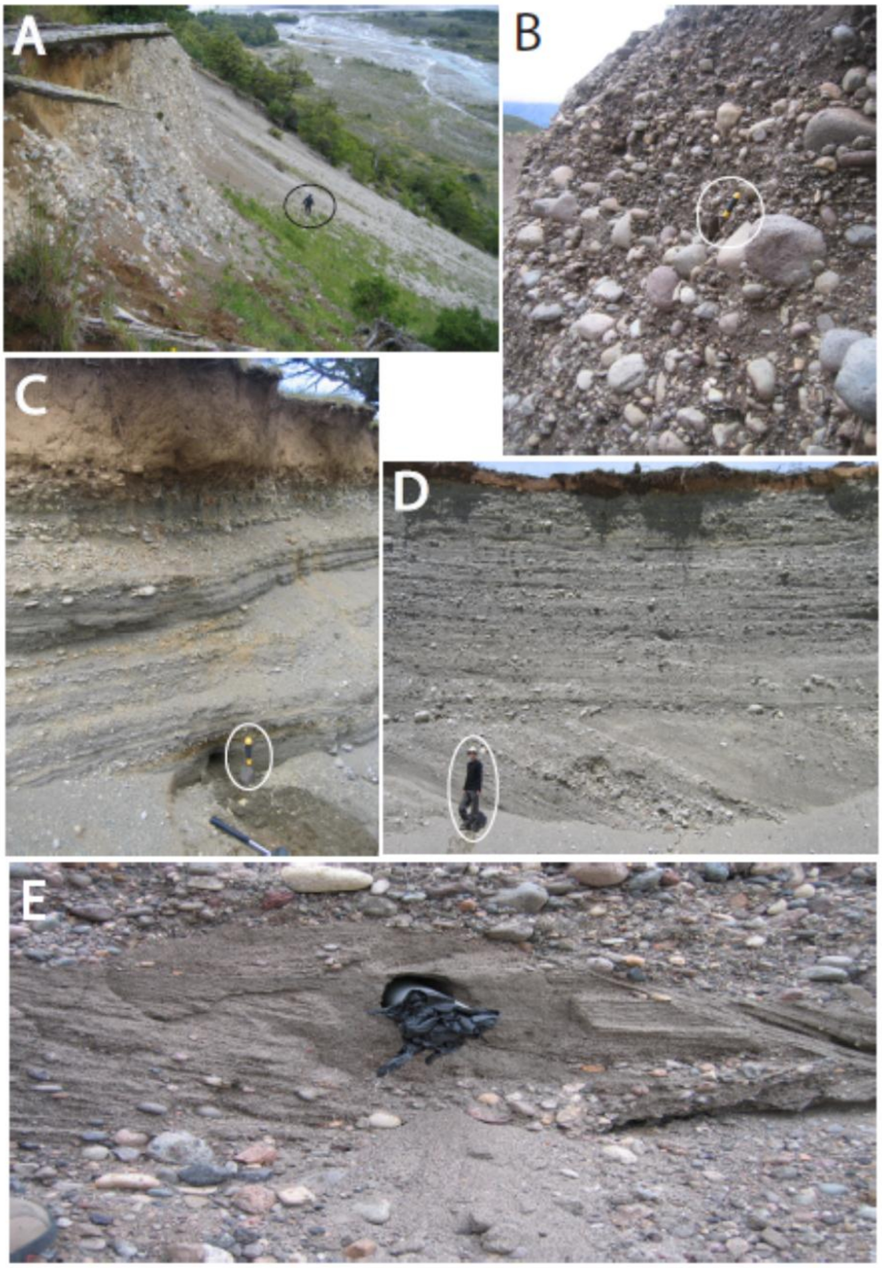

Figure S3

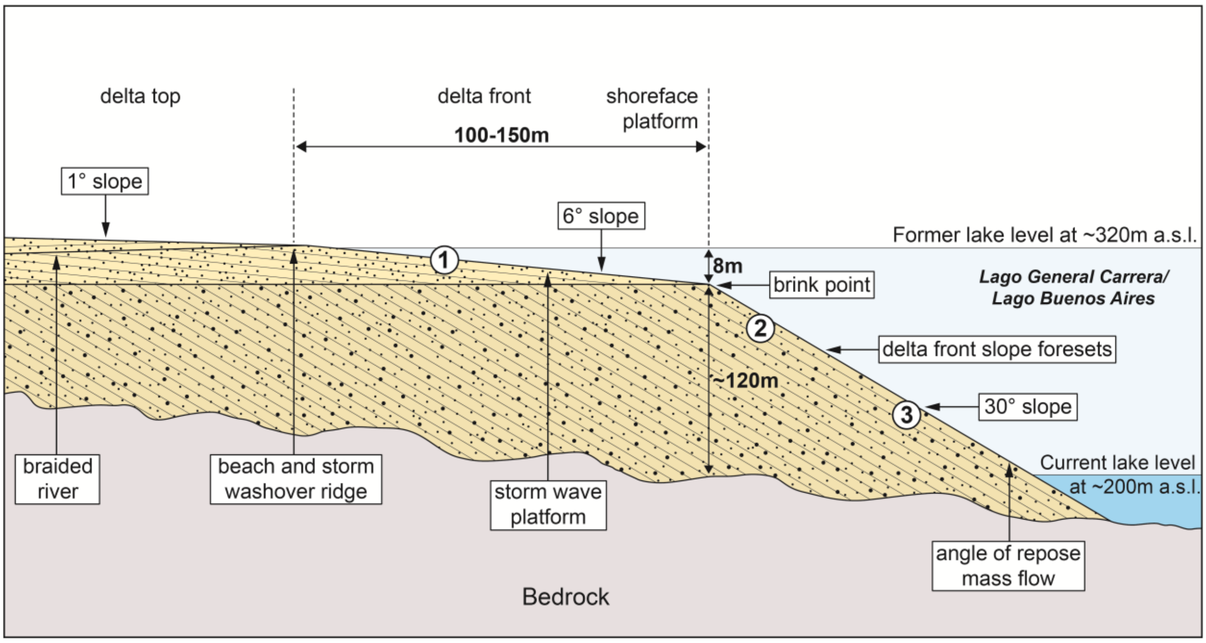

delta top
delta front
shoreface
platform
100-150m
1° slope
6° slope
Former lake level at ~320m a.s.l.
1
8m
brink point
Lago General Carrera/
Lago Buenos Aires
2
delta front slope foresets
~120m
3
30° slope
Current lake level
at ~200m a.s.l.
braided
river
beach and storm
washover ridge
storm wave
platform
angle of repose
mass flow
Bedrock

Figure S4

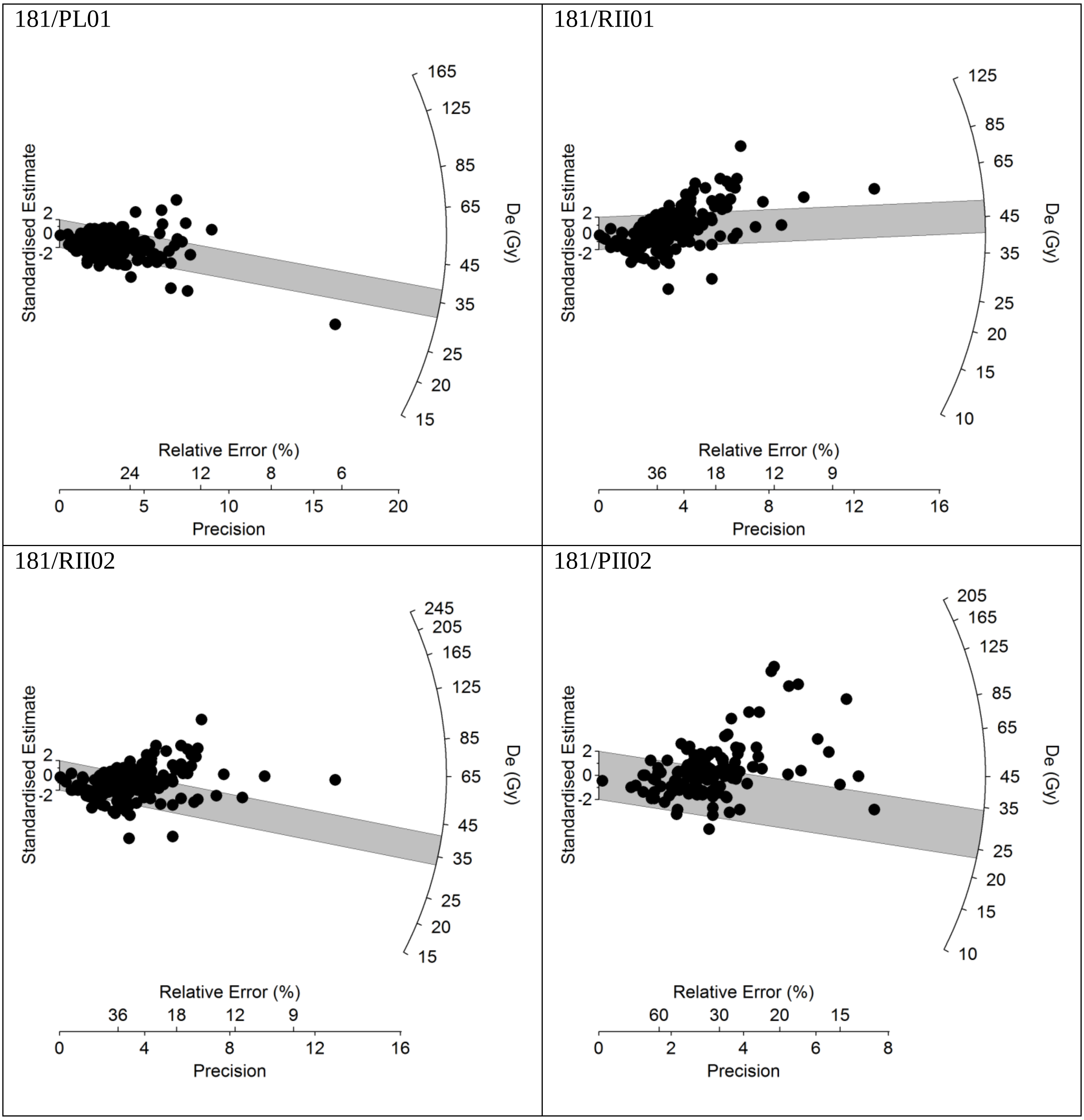
181/PL01
Standardised Estimate
De (Gy)
Relative Error (%)
Precision
181/RII01
Standardised Estimate
De (Gy)
Relative Error (%)
Precision
181/RII02
Standardised Estimate
De (Gy)
Relative Error (%)
Precision
181/PII02
Standardised Estimate
De (Gy)
Relative Error (%)
Precision

Figure S4 Continued

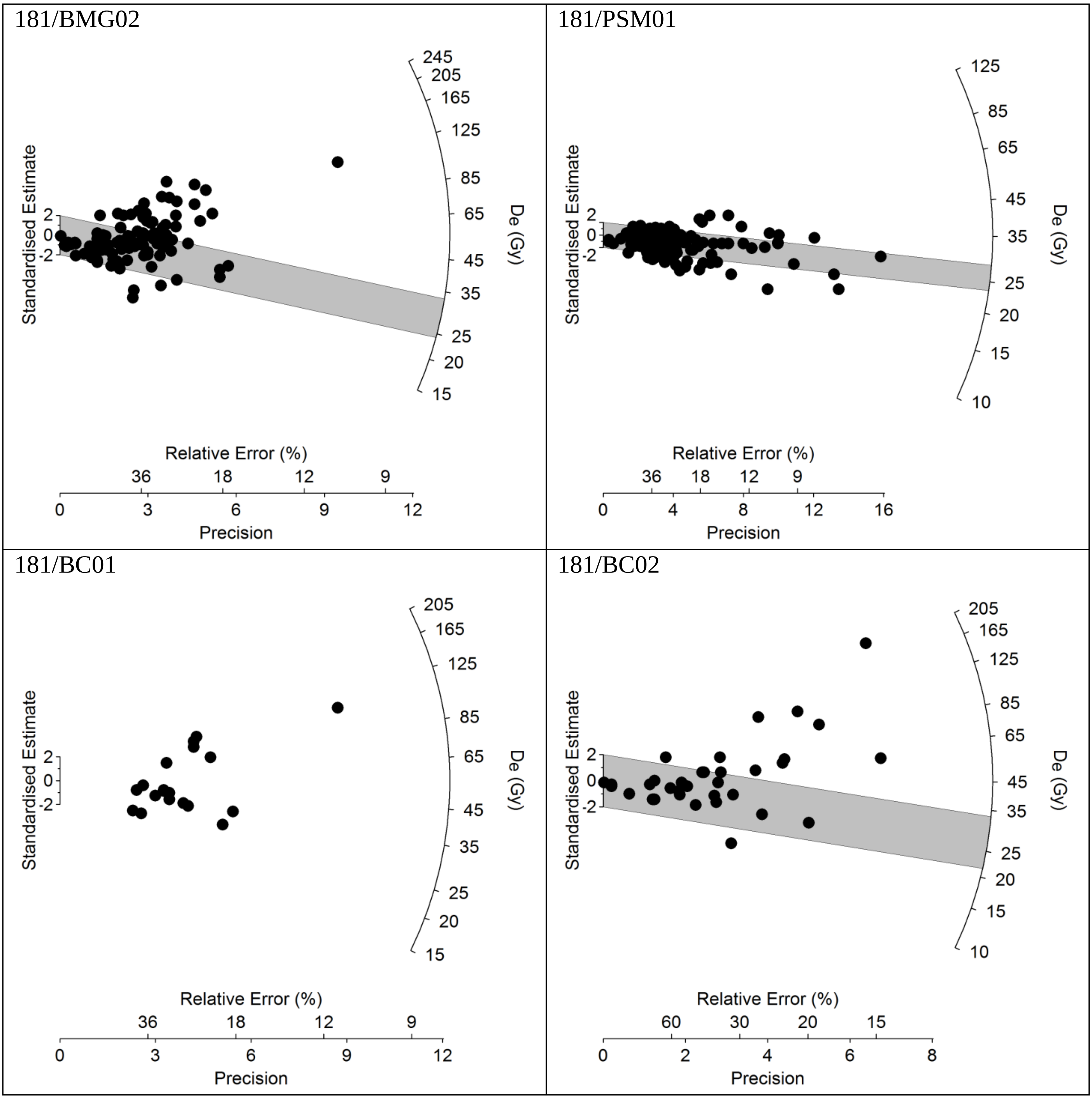

Figure S4 Continued

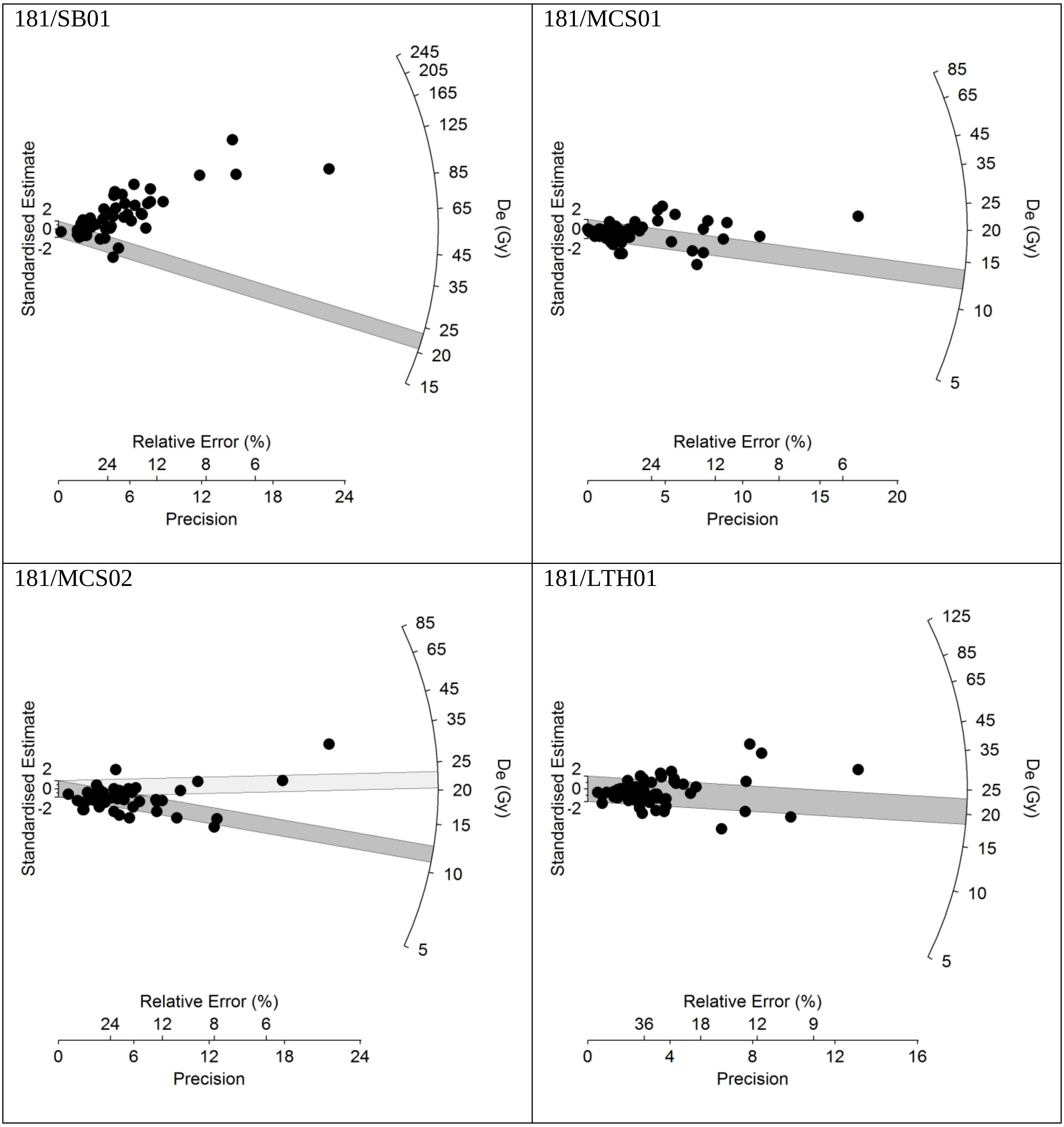

Figure S4 Continued

181/FD02

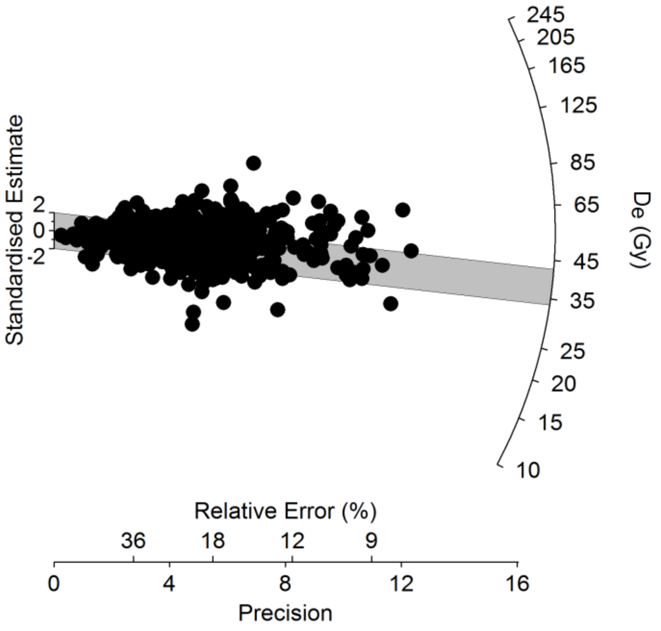

181/JV02

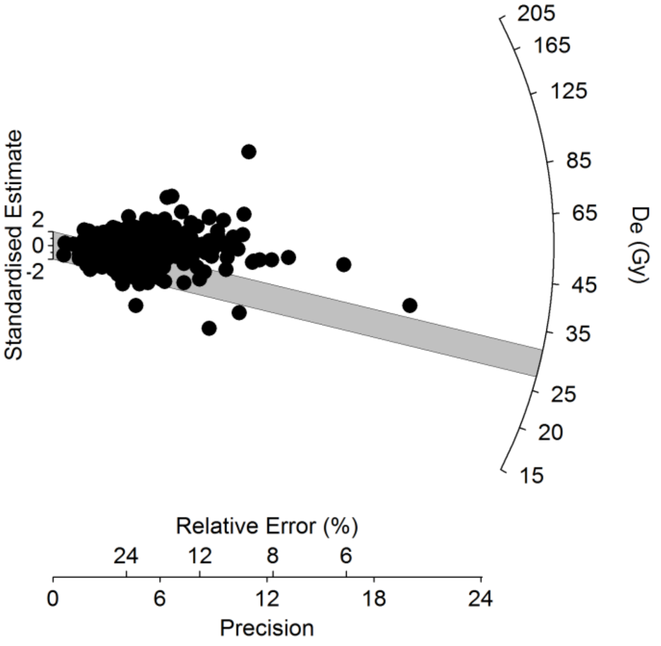

181/WCC01

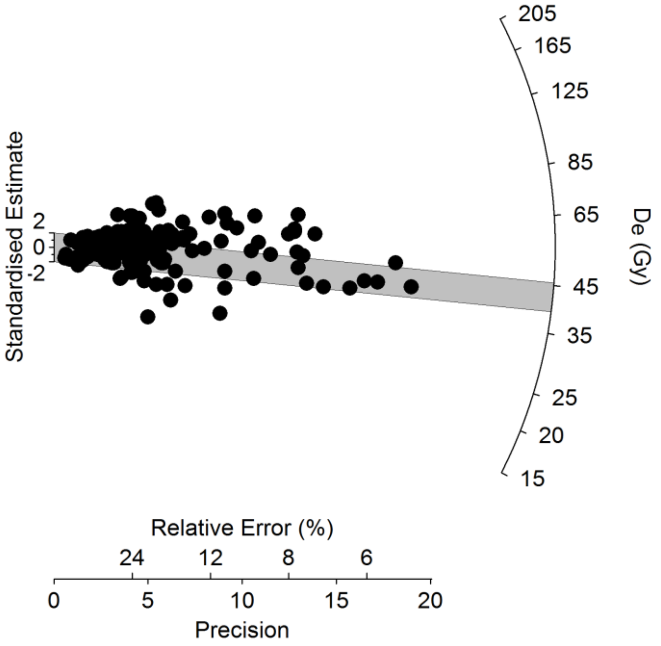

181/LD01

Figure S5

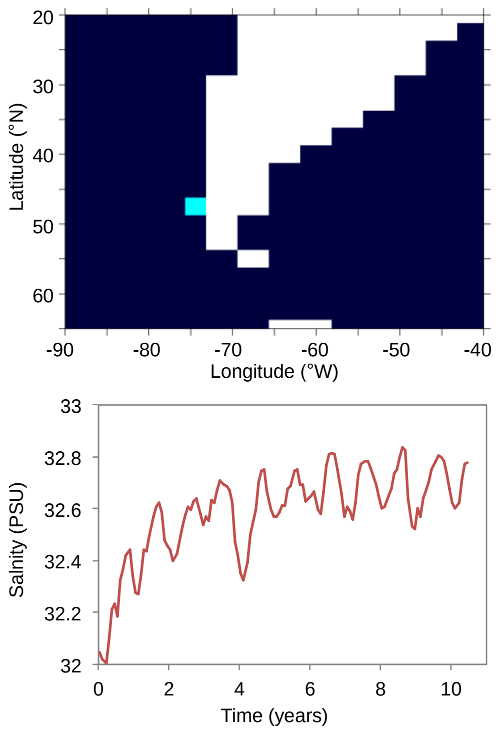

20
30
40
50
60
Latitude (°N)
-90
-80
-70
-60
-50
-40
Longitude (°W)
33
32.8
32.6
32.4
32.2
32
Salinity (PSU)
0
2
4
6
8
10
Time (years)

Table S1

Sample	Given Dose (Gy)	Number of grains	Ratio (Recovered/ Given dose)	Overdispersion (%)
181/FD02	44.6	564 (2000)	1.00 ± 0.02	26
181/JV02	44.0	122 (2000)	1.00 ± 0.03	18
181/WCC01	36.1	122 (2000)	1.04 ± 0.02	14

Table S2

Sample	Depth (m)	Grain size (µm)	Beta Dose (Gy/ka)	Gamma Dose (Gy/ka)	Cosmic Dose (Gy/ka)	Total Dose (Gy/ka)	Number of grains	Age model	D_e (Gy)	Age (ka)
**460-520 masl lake level**										
181/LTH01	2.0 ± 1.0	150 – 250	0.95 ± 0.06	0.60 ± 0.04	0.16 ± 0.08	1.72 ± 0.11	68 (2000)	CAM	20.3 ± 1.5	11.8 ± 1.2
181/MCS02	0.5 ± 0.2	125 – 212	0.93 ± 0.06	0.65 ± 0.05	0.21 ± 0.08	1.79 ± 0.11	47 (200)	CAM	11.7 ± 1.1	6.5 ± 0.7 (12.0 ± 1.3)
**370-440 masl lake level**										
181/BC02	5.0 ± 1.5	180 – 250	1.19 ± 0.07	0.77 ± 0.06	0.12 ± 0.09	2.08 ± 0.13	34 (4000)	FMIX	26.9 ± 4.0	12.9 ± 2.1
181/RII01	3.0 ± 1.0	150 – 250	2.73 ± 0.17	1.58 ± 0.10	0.15 ± 0.08	4.45 ± 0.21	168 (1400)	FMIX	45.7 ± 3.3	10.3 ± 0.9
181/PII02	6.0 ± 1.0	180 – 212	1.50 ± 0.06	0.94 ± 0.06	0.10 ± 0.05	2.54 ± 0.10	133 (2000)	FMIX	28.2 ± 11	11.1 ± 4.2
181/WCC01	3.0 ± 1.0	150 – 250	1.48 ± 0.09	0.89 ± 0.06	0.15 ± 0.08	2.52 ± 0.13	178 (2000)	FMIX	42.2 ± 3.0	16.8 ± 1.5
181/BC01	8.0 ± 1.5	150 – 212	1.07 ± 0.07	0.66 ± 0.05	0.09 ± 0.06	1.82 ± 0.10	19 (4000)	-	-	-
**300-320 masl lake level**										
181/PL01	4.0 ± 1.0	150 – 250	2.04 ± 0.13	1.26 ± 0.08	0.13 ± 0.07	3.43 ± 0.16	210 (1400)	FMIX	34.9 ± 1.6	10.2 ± 0.7
181/RII02	5.0 ± 1.0	150 – 212	2.42 ± 0.15	1.34 ± 0.09	0.12 ± 0.06	3.88 ± 0.18	248 (2000)	FMIX	36.7 ± 2.6	9.5 ± 0.8
181/MCS01	3.0 ± 1.0	180 – 212	0.75 ± 0.05	0.62 ± 0.05	0.15 ± 0.08	1.52 ± 0.10	67 (4000)	CAM	12.9 ± 1.4	8.5 ± 1.1
181/BMG02	10.0 ± 2.0	180 – 250	1.59 ± 0.06	1.03 ± 0.07	0.07 ± 0.07	2.69 ± 0.11	103 (3900)	FMIX	28.4 ± 2.0	10.6 ± 0.9
181/FD02	1.0 ± 0.5	180 – 212	2.03 ± 0.12	1.20 ± 0.09	0.19 ± 0.05	3.42 ± 0.16	726 (2000)	FMIX	37.7 ± 1.7	11.0 ± 0.7
181/JV02	4.0 ± 1.0	180 – 212	1.75 ± 0.11	1.11 ± 0.08	0.13 ± 0.07	3.00 ± 0.15	594 (2000)	FMIX	29.4 ± 1.5	9.8 ± 0.7
181/PSM01	1.0 ± 0.5	150 – 212	1.84 ± 0.11	1.22 ± 0.09	0.19 ± 0.05	3.24 ± 0.15	141 (2000)	CAM	25.8 ± 1.0	8.0 ± 0.5
181/SB01	3.0 ± 1.0	150 – 212	1.13 ± 0.07	0.76 ± 0.06	0.15 ± 0.08	2.04 ± 0.12	49 (3100)	FMIX	22.2 ± 2.9	10.9 ± 1.6
181/LD01	0.0 ± 0.5	150 – 250	1.35 ± 0.08	0.81 ± 0.06	0.30 ± 0.08	2.46 ± 0.13	55 (3200)	CAM	0.39 ± 0.40	0.16 ± 0.16

Notes:

1. The dose rate to all samples was calculated assuming an average water content of 20±5% during the period of burial

Table S3

Stage	Age (kyr)	Glacial lake	Elevation	Area (km^2)	Volume (km^3)	Drainage volume (km^3)	Drainage route	Outlet/col	Destination of meltwater
**A**	Early stages of Glacial Lake LC/LP and LGC/LBA were formed. Delta features as high as 700 and 550 respectively indicate the existence of these stages. Glacial Lake Chacabuco existed as an isolated lake which was drained into Glacial lake LC/LP. These glacial lakes were successively lowered by moraine failure eat of the major basins.								
	**?**	**Glacial Lake LC/LP**	700	*3518-4660	*877-1218		Successive lowering by moraine failure east of Lago Cochrane, Cueva de Manos		
	**13.8**	**Glacial lake Chacabuco (Isolated after LC/LP was lowered below 620 m a.s.l.)**	620	#	#			Col at 620 masl southeast of Pasa Roballos (C-1).	Into Glacial Lake LC/LP
			580				Southward towards Puesto Tejuela. Defined channel at 580 masl (Ds-1)	Former drainage route at the col at 580 masl NE of Puesto Tejuela	
	**13.8-11.5**	**Glacial Lake LC/LP**	550				Successive lowering by moraine failure east of Lago Cochrane, Rio Blanco (Ds-2)	Moraine east of Lago Cochrane at the head waters of Rio Blanco (480 masl)	
			480				Canyons along Carretera Austral East Lago Bertrand into LGC basin (Ds-4)		Glacial Lake LGC/LBA
	**13.8-11.5**	**Glacial Lake LGC/LBA**	550	*4068-5546	*847-1202	ˆ1153	Successive lowering by moraine failure east of Lago General Carerra, Rio Deseado (Ds-3)	Adjusted to col east of Lago General Carrera	**Eastward into the Atlantic**
			480	¤6492	¤1267				
**B**	**11.5-10.0**	**Glacial Lake PIS**	480	7354	1461	“649	Successive lowering by moraine failure east of Lago General Carerra, Rio Deseado (Ds-5)	Adjustment to col at 380 masl east of Lago General Carrera (C-2)	**Eastward into the Atlantic**
			380	5448	812				
**C**	**10.0**	**Glacial Lake PIS**	380	5521	829	§161		Col at 380 masl east of Lago General Carrera	**West into the Pacific**
			350	4951	668		Drainage route through canyon at Lago Tranquilo (Ds-6)	Col at 350 masl west Lago Tranquilo (C-3)	
**D**	**8.5**	**Glacial Lake PIS**	350	5533	770	±189	Drainage through col at	Present day col at 315	**Eastward into the Atlantic**

			315	4951	581		the head waters of Rio Bravo north Villa O'Higgins (Ds-7)	masl Intermediate stage at 330 masl possibly caused by erosion of col (C-4)	
**E**	**8.0**	**Glacial Lake PIS**	315	5247	641	[±]271	Drainage through Rio Pascua into Lago O'Higgins (Ds-8)	Present day col at 260 masl (C-5)	**Eastward into the Atlantic**
			260	4385	370				
		**Glacial Lake PIS final drainage**	260	4385	370	370	Drainage into the Golfo Peñas (Ds-9) at the final separation of the Patagonian Ice Sheet		**West into the Pacific**
			200						

* Min and maximum extent.
[#] Included in the LC/LP extent.
[¤] Calculated on the inferred 11.5 ka ice margin.
[^] Calculated on the maximum extent for the highest glacial lake levels.
“ Calculated on the inferred 10.5 ka ice margin.
[§] Calculated on the inferred 9.5 ka ice margin.
[±] Calculation based on the ice margin needed to block the col.
